# Streptolysin O Rapidly Impairs Neutrophil Oxidative Burst and Antibacterial Responses to Group A *Streptococcus*

**DOI:** 10.3389/fimmu.2015.00581

**Published:** 2015-11-16

**Authors:** Satoshi Uchiyama, Simon Döhrmann, Anjuli M. Timmer, Neha Dixit, Mariam Ghochani, Tamara Bhandari, John C. Timmer, Kimberly Sprague, Juliane Bubeck-Wardenburg, Scott I. Simon, Victor Nizet

**Affiliations:** ^1^Department of Pediatrics, University of California San Diego, La Jolla, CA, USA; ^2^Department of Biomedical Engineering, University of California Davis, Davis, CA, USA; ^3^Department of Biological Sciences, San Diego State University, San Diego, CA, USA; ^4^Department of Pharmacology, University of California San Diego, La Jolla, CA, USA; ^5^Department of Pediatrics, University of Chicago, Chicago, IL, USA; ^6^Skaggs School of Pharmacy and Pharmaceutical Sciences, University of California San Diego, La Jolla, CA, USA

**Keywords:** group A *Streptococcus*, *Streptococcus pyogenes*, pore-forming toxin, streptolysin O, neutrophils, oxidative burst, neutrophil extracellular traps, infection

## Abstract

Group A *Streptococcus* (GAS) causes a wide range of human infections, ranging from simple pharyngitis to life-threatening necrotizing fasciitis and toxic shock syndrome. A globally disseminated clone of M1T1 GAS has been associated with an increase in severe, invasive GAS infections in recent decades. The secreted GAS pore-forming toxin streptolysin O (SLO), which induces eukaryotic cell lysis in a cholesterol-dependent manner, is highly upregulated in the GAS M1T1 clone during bloodstream dissemination. SLO is known to promote GAS resistance to phagocytic clearance by neutrophils, a critical first element of host defense against invasive bacterial infection. Here, we examine the role of SLO in modulating specific neutrophil functions during their early interaction with GAS. We find that SLO at subcytotoxic concentrations and early time points is necessary and sufficient to suppress neutrophil oxidative burst, in a manner reversed by free cholesterol and anti-SLO blocking antibodies. In addition, SLO at subcytotoxic concentrations blocked neutrophil degranulation, interleukin-8 secretion and responsiveness, and elaboration of DNA-based neutrophil extracellular traps, cumulatively supporting a key role for SLO in GAS resistance to immediate neutrophil killing. A non-toxic SLO derivate elicits protective immunity against lethal GAS challenge in a murine infection model. We conclude that SLO exerts a novel cytotoxic-independent function at early stages of invasive infections (<30 min), contributing to GAS escape from neutrophil clearance.

## Introduction

The Gram-positive bacterium group A *Streptococcus* (GAS; *Streptococcus pyogenes*) is a leading human pathogen ranked among the top 10 causes of infection-associated mortality worldwide (1). An estimate of 5–15% of all humans are colonized with GAS, which can cause a wide spectrum of infections ranging from self-limiting pharyngitis and impetigo to invasive and life-threatening diseases including streptococcal toxic shock syndrome and necrotizing fasciitis (2). Among 700 million GAS infections worldwide annually, an estimated 1.8 million severe infections occur with a mortality rate as high as 25% (1). A commercial vaccine to protect against GAS infections is not yet available (3).

The ability of GAS to produce serious disease, sometimes in previously healthy children and adults, defines a capacity of the organism to resist clearance by frontline elements of the host innate immune system, including neutrophils, the most abundant circulating leukocytes, which are rapidly recruited to sites of bacterial infection (4, 5). GAS express an array of virulence factors that allow the bacterium to counteract neutrophil processes and molecular effectors of bactericidal activity (6), including the anti-phagocytic surface hyaluronic acid capsule and M protein (7, 8), nuclease-mediated degradation of neutrophil extracellular traps (NETs) (9, 10), factors that neutralize reactive oxygen species (ROS) (11), and inhibitors of neutrophil-derived cationic antimicrobial peptides (12). A global increase in invasive GAS infections has been attributed to the rise of a single, globally disseminated GAS M1T1 clone (13, 14). GAS M1T1 strains can undergo a spontaneous mutation in the two component global regulatory system *covR/S* (also known as *csrR/S*) *in vivo* leading to upregulation of capsule, nuclease, and other GAS virulence factors (15, 16), and neutrophil resistance may be an important selection pressure for the survival and bloodstream dissemination of these mutants (17).

In addition to these “defensive” survival properties, GAS is remarkable for its potential to induce the apoptotic cell death of neutrophils (18), a phenotype that was subsequently linked to the bacterium’s production of the potent pore-forming toxin, streptolysin O (SLO) (19). The *slo* gene has universally been found to be present in the genome of all GAS serotypes and strains, and encodes a toxin belonging to the cholesterol-dependent cytolysins (CDCs), a family comprising at least 28 members/bacterial species, including *Streptococcus pneumoniae* pneumolysin and *Listeria monocytogenes* listeriolysin O (20). SLO is a multi-functional protein with pore-dependent and -independent functions (21, 22). SLO is highly expressed by epidemic M1 GAS strains (23), and is further upregulated upon *covR/S* mutation in the GAS M1T1 background (15).

In a general sense, SLO toxicity to neutrophils has been hypothesized to contribute to GAS bloodstream survival and virulence (24, 25). Given the strong bioactivities and high expression of this toxin, as well as its prominence in invasive M1T1 GAS infection, we undertook a mechanistic analysis of its impact on specific neutrophil functions and bactericidal activity. We discovered that SLO has the capacity to suppress neutrophil oxidative burst and other key neutrophil functions including degranulation, directed migration, and formation of NETs, together promoting neutrophil resistance. Immunization with an inactivated form of SLO (rSLOmut) induced high titers of neutralizing IgG and IgM anti-SLO antibodies affording passive protection in a mouse model of systemic GAS infection, confirming its attractiveness as a component of a future multicomponent vaccine against this pathogen.

## Materials and Methods

### Bacterial Strains

Group A *Streptococcus* wild-type (WT) strain M1T1 5448 was originally isolated from a patient with necrotizing fasciitis and toxic shock syndrome (26). The isogenic M1T1 5448 ΔSLO mutant and complemented (ΔSLO + pSLO) strain were described previously (19). The animal-passaged (AP) version of the M1T1 GAS parent strain contains a single inactivating adenine insertion at the 877-bp position of *covS* (16). A panel of 33 GAS isolates of various M serotypes and clinical associations was provided from collections of U.S. Centers for Disease Control and Prevention (CDC). Additional strains used were *S. pneumoniae* D39, *Staphylococcus aureus* Newman strain, and methicillin-resistant *S. aureus* (MRSA) TCH1516 WT and its isogenic ΔHla mutant. Bacteria were cultivated in Todd–Hewitt broth (THB) at 37°C.

### Isolation of Human and Murine Neutrophils

Neutrophils were isolated from freshly collected whole blood of healthy donors under a protocol approved by the University of California San Diego (UCSD) Human Research Protections Program, using PolyMorphPrep Kit (Fresenius Kabi), as previously described (27). Mice were sacrificed and blood was collected via terminal cardiac puncture under a protocol approved by the UCSD Institutional Animal Care and Use Committee. Mouse blood was diluted fivefold in phosphate-buffered saline (PBS, Cellgro) and neutrophils were isolated by layers of Histopaque-1077 and Histopaque-119 (Sigma). Remaining red blood cells (RBCs) were eliminated in lysis buffer (0.15M NH_4_Cl, 10 mM KHCO_3_, 0.1 mM EDTA).

### Oxidative Burst Assay

Oxidative burst assays were performed as previously described (28). Briefly, 2 × 10^6^/mL human or 2 × 10^5^/mL mouse neutrophils were loaded with 20 μM 2,7-dichlorofluorescein diacetate (DCFH-DA; Fisher) in Hank’s balanced salt solution (HBSS, Cellgro) without Ca^2+^ and Mg^2+^ and incubated with rotation at 37°C for 20 min. Neutrophils were resuspended to a density of 1 × 10^7^ cells/mL and 1 × 10^6^ cells/well were infected at a multiplicity of infection (MOI) = 1 bacteria/cell and incubated for 20 min at 37°C/5% CO_2_. Assays were performed using untreated control (control), 25 nM phorbol 12-myristate 13-acetate (PMA, Sigma) as positive control, 10 μM diphenyleneiodonium (DPI, Sigma) to block generation of ROS, or recombinant SLO protein (rSLO), anti-SLO antibody (Abnova), or water-soluble cholesterol (Sigma) at the stated concentrations. The fluorescence intensity was quantified on a SpectraMax M3 plate reader at 485 nm excitation/520 nm emission using SoftMax Pro software.

### Neutrophil Killing Assays

Neutrophils were isolated as described above, and 1 × 10^6^ cells in 100 μL were challenged with bacteria at MOI = 10 bacteria/cell at 37°C for 30 min with rotation. To pinpoint extracellular killing, cells were treated with 10 μg/mL cytochalasin D (cyt. D, Sigma) for 10 min prior to infection to inhibit phagocytosis, in the presence or absence of 500 mU/mL desoxyribonuclease (DNase, Sigma) to evaluate the contribution of DNA-based NETs to bacterial killing. Surviving colony-forming units (CFU) for all killing assays were enumerated by Todd-Hewit agar (THA) dilution plating. Survival was calculated as the percentage of the initial inoculum.

### Red Blood Cell Hemolysis Assay

Red blood cells were isolated from human blood and supernatants from bacterial cultures at various growth phases OD_600_ = 0.3–0.7, and tested for hemolysis activity of secreted SLO. In these assays, supernatant was incubated with 4 mM dithiothreitol (DTT) to stabilize SLO and 0.0004% trypan blue at room temperature (RT) for 10 min to inhibit streptolysin S activity. RBCs were incubated with bacterial culture supernatant with or without anti-SLO antibodies or water-soluble cholesterol (Sigma) as indicated for 30 min at 37°C, with PBS (0% hemolysis) or H_2_O (100% hemolysis) as positive and negative controls. Supernatants were collected from assay wells after centrifugation at 3000 × *g* for 15 min, and hemolysis determined by absorbance with a SpectraMax M3 plate reader at 541 nm using SoftMax Pro software. Each titer was recorded as the point that the hemolysis reached half of the 100% RBC lysis (H_2_O) control.

### Neutrophil Elastase and IL-8 Release

Neutrophils were infected with bacteria at an MOI of 1 for 30 min as described earlier. The release of neutrophil elastase in response to bacteria was indirectly determined via the proteolytic activity of elastase. Cell-free supernatant was incubated with 20 μM of peptide substrate *N*-(Methoxysuccinyl)-Ala-Ala-Pro-Val 4-nitroanilide (Sigma) for 20 min at RT. The absorbance was measured with SpectraMax M3 plate reader at 405 nm for elastase using SoftMax Pro software. IL-8 release from neutrophils was quantified using the Quantikine ELISA kit (R&D Systems) following the manufacturer’s manual. Briefly, 1 × 10^6^ neutrophils were infected with live GAS WT and ΔSLO at an MOI of 1 for 60 min and the release of IL-8 was determined via absorbance with SpectraMax M3 plate reader at 450 nm using SoftMax Pro software.

### Neutrophil Migration

Migration distance was measured by perfusing neutrophils with GAS WT, ΔSLO mutant bacteria at an MOI of 10 and 50. Five nM IL-8 was used as a chemotactic stimulus. Migration of individual neutrophils was quantified by measuring the distance in micrometer after 3 min compared to starting point by microscopy (Zeiss Axiolab).

### Immunostaining of NETs and NET Quantification

Neutrophil extracellular trap visualization was performed as described previously (29). Briefly, 5 × 10^6^ neutrophils were infected with GAS WT and ΔSLO mutant was added at MOI = 1 and incubated for 4 h in 37°C/5% CO_2_. Cells were fixed with 4% paraformaldehyde and stained with anti-myeloperoxidase (MPO) antibody (1:300 diluted, Calbiochem) in PBS + 2% bovine serum albumin (BSA, Sigma) for 1 h at RT, followed by incubation with goat anti-rabbit Alexa 488 antibody (1:500 dilution, Life Technologies). Cells were counterstained with ProlongGold + 4′,6′-diamidino-2-phenylindole (DAPI, Invitrogen) and imaged on a fluorescent microscope. Representative, randomized images (*n* = 5) were taken for each condition and individual experiment. The ratio of NET-releasing cells to non-NET-releasing cells was determined as % of total cells releasing NETs.

### Transmission Electron Microscopy

Neutrophils were infected with GAS WT and ΔSLO mutant for 4 h at an MOI of 1 and cells were prepared for transmission electron microscopy (TEM) as previously described (19, 30). Briefly, cells were fixed with 3% formaldehyde, 1.5% glutaraldehyde, 0.1M sodium cacodylate trihydrate, 5 mM CaCl_2_ and 2.5% sucrose at pH 7.4 buffer for 1 h. Cells were washed three times in ice-cold 0.1M sodium cacodylate buffer containing 2.5% sucrose and fixed cells were incubated with Palade’s OsO_4_ by incubation with 1% osmium tetroxide in acetate/veronal solution for 1 h on ice. After three washing with acetate/veronal solution, the cells were stained and stabilized *en bloc* in 0.5% uranyl acetate with acetate/veronal solution overnight at RT in the dark. After one rinse with ddH_2_O and one rinse with ice-cold 50% ethanol, the cells were dehydrated in an ice-cold dehydration series of 70, 95, and 100% ethanol for 15 min followed by three washes in 100% acetone at RT. Cells were then infiltrated in well-mixed Epon-ethanol resin series of three: 3% for 7 h, 66% for 7 h, followed by 100% at least overnight with agitation at RT. Next, the samples were allowed to polymerize in 100% Epon blocks at 60–80°C for a minimum of 48 h. Thin sections (70 nm) were examined with FEI Tecnai 12 transmission electron microscope and images taken with Tietz 214 CCD camera. Images were adjusted in brightness and contrast with Adobe Photoshop CS version 8.

### Generation of Inactivated rSLOmut

Based on the SLO crystal structure (31), a recombinant SLO derivate (rSLOmut) with mutated tryptophan residue in the membrane-binding loop W535A was generated similarly as described (32). Loss of hemolytic activity of the rSLOmut protein was determined in J774 murine macrophages by TUNEL assay as described (19). The rSLOmut protein was cloned by overlapping PCR into a pET15b expression vector and expressed in *Escherichia coli* BL21 by induction at OD_600_ 0.6–1.0 with 1 mM isopropyl-beta-d-thiogalactopyranoside (IPTG) for 4 h. Protein purification and quantification by A_280_ was performed as described (19).

### SLO Immunization Study

Eight-week-old male CD1 mice (Charles River Laboratories) were immunized three times, 2 weeks apart, with recombinant SLOmut protein or PBS control, plus adjuvant. The first immunization was done with Freund’s complete adjuvant, and the following two immunizations were done with Freund’s incomplete adjuvant. Protein or PBS were mixed with adjuvant 1:1 and emulsified, resulting in a final protein concentration of 500 μg/mL. Each mouse was injected intraperitonealy (i.p.) with 100 μL of the protein/adjuvant emulsified mixture. Mice were infected i.p. with 8 × 10^7^ CFU bacteria 2 weeks after the last immunization. Survival was monitored daily for 14 days. Anti-SLO IgG and IgM antibody titer was measured by ELISA using the serum collected 3 days before challenging the mice with GAS.

### Statistical Analysis

All data were collected from at least three independent experiments in triplicate. Experiments using neutrophils were performed with a minimum of three different healthy volunteers. The data were combined and expressed as mean ± SEM except stated differently. All data were analyzed by unpaired Student’s *t*-test using GraphPad Prism version 5.0 (GraphPad Software Inc.). *P* values <0.05 were considered statistically significant.

## Results

### GAS Production of SLO Suppresses Neutrophil Oxidative Burst

**Figure 1:**
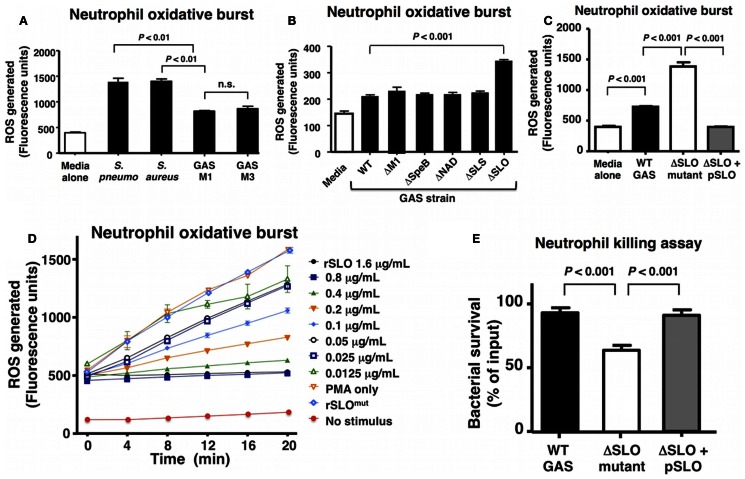
**Group A *Streptococcus* SLO suppresses neutrophil oxidative burst**. **(A)** GAS WT serotype M1 and M3 suppress oxidative burst response in human neutrophils compared to representative WT strains of *Streptococcus pneumoniae* (D39) and *Staphylococcus aureus* (Newman) and media only control. **(B)** An isogenic GAS ΔSLO mutant fails to suppress oxidative burst in neutrophils compared to isogenic ΔM1, ΔSpeB, ΔNAD, and ΔSLS mutants and GAS WT. **(C)** The complemented GAS ΔSLO mutant (ΔSLO + pSLO) restores the suppression of oxidative burst. **(D)** Recombinant, active SLO protein suppresses oxidative burst in a dose-dependent manner in PMA-stimulated neutrophils, including media alone as a negative control. **(E)** SLO protects against killing by neutrophils at an MOI of 10 at 30 min post infection. Results are given in average ± SEM and analyzed by Student’s *t*-test (n.s., not significant).

A select group of Gram-positive human pathogens including GAS, *S. aureus*, and *S. pneumoniae* are leading causes of invasive, potentially life-threatening infections worldwide (1, 33, 34). Neutrophils are a critical first line of defense against such invading pathogens, and the rapid production of bactericidal ROS is key element of their effectiveness. Compared to *S. aureus* and *S. pneumoniae*, GAS of the M1 and M3 serotypes elicited a reduced level of oxidative burst from freshly isolated human neutrophils 20 min post infection (Figure 1A). As each bacterial species is replete with pathogen-associated molecular patterns (e.g., lipoteichoic acid, peptidoglycan, and formyl peptides) to stimulate neutrophil activation, we hypothesized that GAS produced factor(s) that suppressed the full host oxidative burst potential. By screening a panel of isogenic M1T1 GAS mutants lacking defined virulence factors, we identified a higher neutrophil oxidative burst in response to a GAS ΔSLO mutant, suggesting that the toxin could play a key role in oxidative burst suppression (Figure 1B).

Streptolysin O is a well-studied, secreted virulence factor of GAS that induces apoptosis in neutrophils and macrophages at later time points (~4 h) (19). We, thus, focused the aim of the current study to investigate the role of SLO in the early GAS–neutrophil interaction (<30 min) independent of cell death. Incubation of human neutrophils with GAS WT or ΔSLO mutant at MOI of 1, 10, or 50 bacteria/cell for 30 min did not produce cell death as assessed by LDH release (Figure S1A in Supplementary Material) or Live–Dead staining (Figures S1B,C in Supplementary Material). Complementation of the ΔSLO mutant with the *slo* gene expressed on a plasmid vector (ΔSLO + pSLO) restored the WT phenotype confirming that SLO dampens the neutrophil ROS response (Figure 1C). Additionally, purified recombinant SLO (rSLO) protein was sufficient to reduce the oxidative burst in neutrophils in a dose-dependent manner to the levels of the untreated control (Figure 1D). Oxidative burst suppression was not a universal property of bacterial pore-forming toxins, as live methicillin-resistant *S. aureus* (MRSA) USA300 producing the pore-forming virulence factor α-hemolysin (Hla) did not differ from an isogenic ΔHla mutant in elicitation of neutrophil ROS (Figure S2A in Supplementary Material), nor did recombinant Hla toxin at concentrations between 10 and 1000 ng/mL modulate oxidative burst (Figure S2B in Supplementary Material).

As ROS production is an important bactericidal effector of neutrophils, we confirmed the overall effect of SLO on neutrophil bacterial killing. The ΔSLO mutant was significantly more susceptible to rapid neutrophil killing after 30 min of infection than the M1T1 GAS parent strain, and WT resistance was restored in the complemented mutant (Figure 1E).

### GAS M1 Serotype and *covRS* Mutation Are Associated with Higher SLO Activity

**Figure 2:**
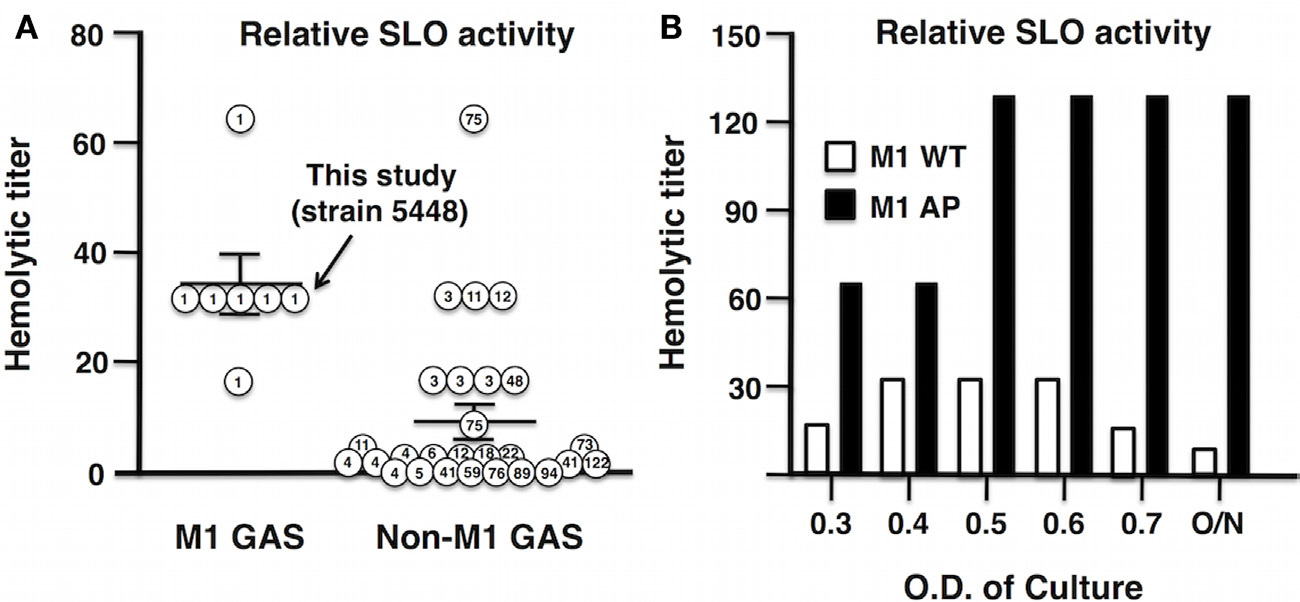
**Streptolysin O activity is enhanced in GAS M1 serotype and further enhanced after AP of GAS M1T1 clone**. **(A)** Thirty-three clinical GAS isolates from the CDC were grouped into M1 serotype and non-M1 serotypes, where M1 serotypes including M1 5448 show increased hemolytic activity compared to non-M1 serotypes. **(B)** Hemolytic activity of SLO in GAS WT peaks in log-phase, whereas in an AP strain, M1 is elevated and reaches maximal hemolytic activity OD_600_ ≥ 0.5.

An increase in the production of SLO played a key role in the evolution of the globally disseminated M1T1 epidemic clone responsible for GAS infections of increased frequency and severity (13, 14, 23). Analysis of 33 clinical isolates obtained from the U.S. Center for Disease Control and Prevention (CDC) revealed a higher median hemolytic activity of M1 serotypes compared to non-M1 serotypes (Figure 2A). In this study, we utilized GAS strain 5448, a representative of the globally disseminated M1T1 clone, which is the most frequently isolated clone from patients suffering invasive infections (35, 36). During invasive infections in humans, M1T1 GAS (15); and isolates of certain additional serotypes (37) undergo spontaneous mutations in the CovRS regulator, leading to upregulation of virulence factors including SLO and hyaluronic acid capsule, coupled with loss of cysteine protease SpeB expression, which can degrade SLO during stationary phase (38). This “genetic switch” is recapitulated by AP of M1T1 GAS in mice (15, 38). Consistent with this model, we found that the SLO activity of WT M1T1 GAS is maximal in log-phase (OD_600_ 0.4–0.6) and declines in stationary phase, while its expression is markedly increased in the AP strain bearing a *covS* mutation, remaining high through late-log phase and stationary phase due to SpeB inactivation (Figure 2B).

### Inhibitors of SLO Activity Increase the Oxidative Burst Response of Neutrophils to GAS

**Figure 3:**
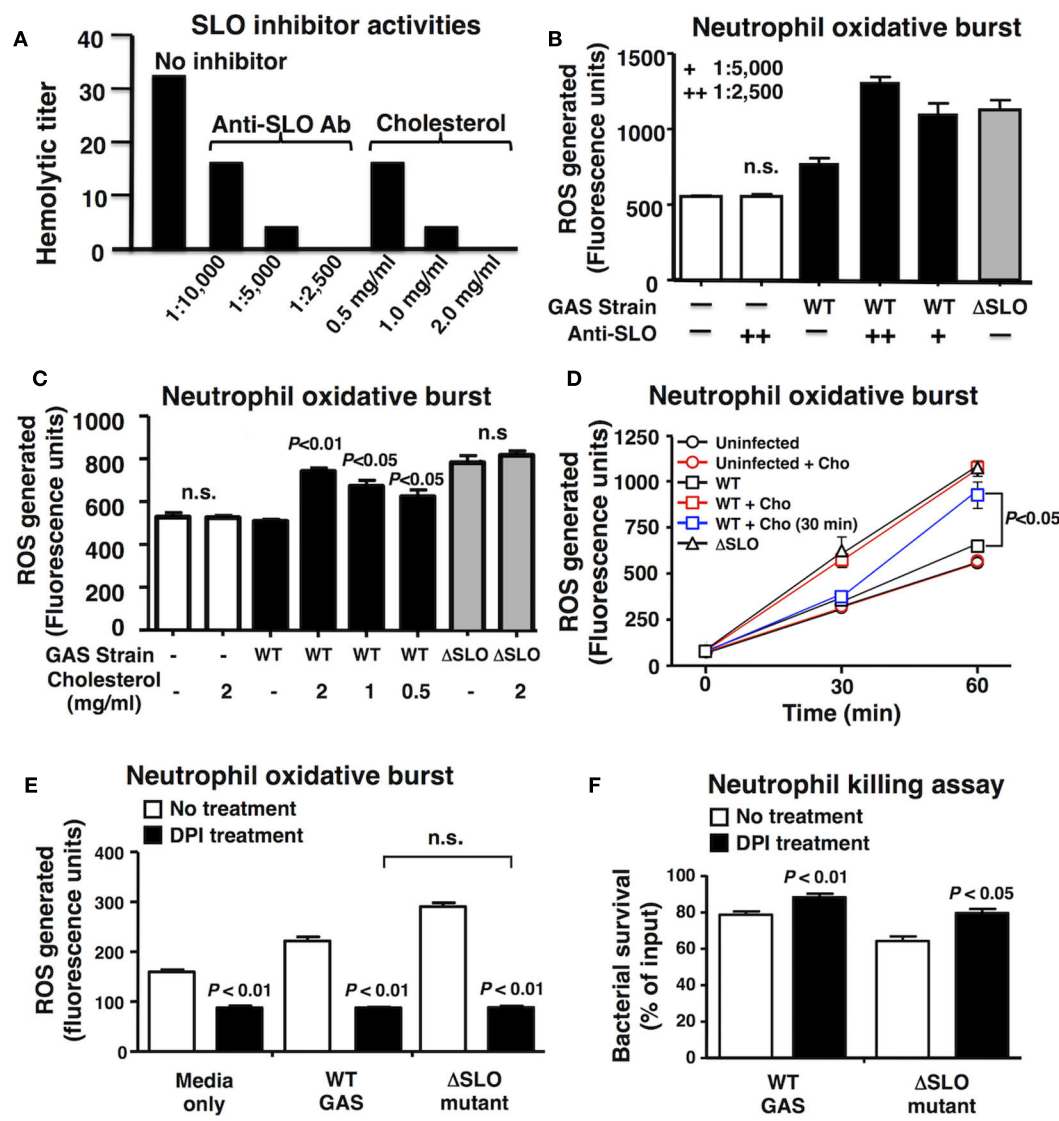
**Inhibition of SLO reverses the suppression of oxidative burst**. **(A)** Anti-SLO antibodies and water-soluble cholesterol block hemolytic activity of SLO in a dose-dependent manner compared to control without inhibitor. **(B)** Inhibition of SLO with anti-SLO blocking antibodies or **(C)** cholesterol prevents suppression of oxidative burst in neutrophils in WT GAS with no effect on ΔSLO mutant or media only control 20 min post infection. **(D)** Oxidative burst in neutrophils infected with bacteria was monitored over a 60 min time course and cholesterol (cho) added after 30 min reversed the suppression by SLO. Effect of pharmacological inhibition of NADPH oxidase activity by DPI compared to untreated control in response to live GAS WT and ΔSLO mutant on **(E)** neutrophil oxidative burst and **(F)** neutrophil killing. Results are given in average ± SEM and analyzed by Student’s *t*-test (n.s., not significant).

To confirm a key role of SLO in suppression of neutrophil oxidative burst, we utilized anti-SLO neutralizing antibodies and water-soluble cholesterol, a known inhibitor of SLO activity (39). Dose-dependent activity of each inhibitor against SLO was confirmed by complete ablation of hemolytic activity (Figure 3A). Both anti-SLO antibodies and cholesterol reversed the suppressive effect of SLO on neutrophil oxidative burst in dose-dependent manners (Figures 3B,C). Interestingly, delayed administration of cholesterol to neutrophils 30 min after exposure to WT GAS restored oxidative burst expression as measured at 60 min (Figure 3D), indicating the effects of the toxin are at least partially reversible upon SLO inhibition. When the pharmacological NADPH oxidase inhibitor, DPI was used to block neutrophil ROS generation (40), the survival difference between the WT and ΔSLO mutant GAS strains was no longer observed (Figure 3E); DPI treatment enhanced survival of both WT and ΔSLO strains, demonstrating that ROS contributes to neutrophil killing of GAS (Figure 3F).

### SLO Suppresses Neutrophil Degranulation and IL-8 Release/Responsiveness

**Figure 4:**
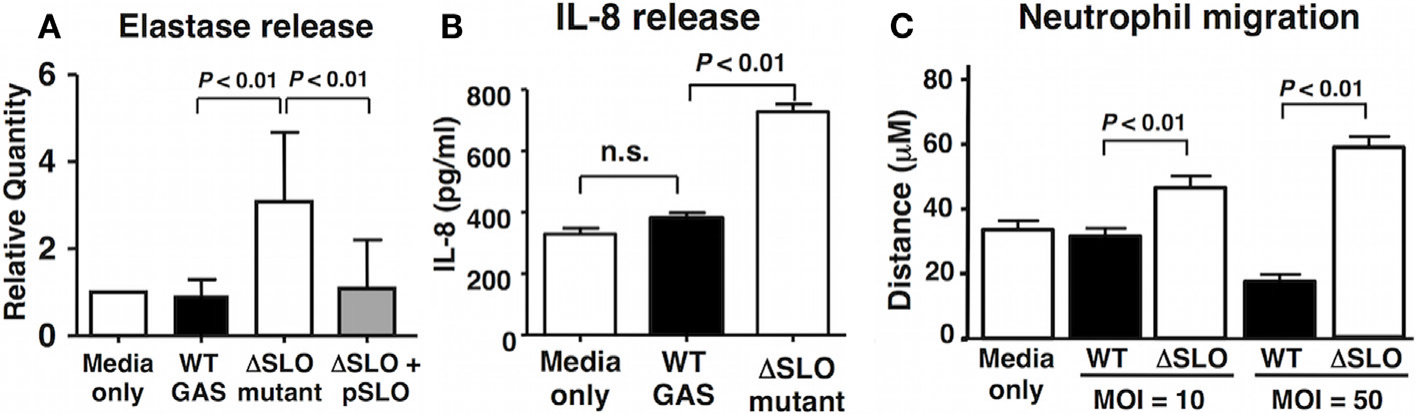
**Streptolysin O inhibits neutrophil degranulation and IL-8 release/responsiveness**. **(A)** Neutrophil degranulation was determined by elastase release upon infection with live GAS WT, ΔSLO mutant, complemented bacteria and media only control, as quantified by ELISA. **(B)** SLO prevents IL-8 release from infected neutrophils compared to ΔSLO mutant bacteria and media only control as quantified by ELISA. **(C)** SLO inhibits neutrophil migration in MOI-dependent manner in contrast to ΔSLO mutant bacteria and media only control as measured by microscopic analysis. Results are given in average ± SEM and analyzed by Student’s *t*-test (n.s., not significant).

Reactive oxygen species have been implicated in the activation of neutrophil granule proteases including elastase (41, 42). GAS suppressed the degranulation of neutrophils in a SLO-dependent manner as quantified by elastase release into the supernatant (Figure 4A). Activated neutrophils themselves are an important source of chemokine interleukin-8 (IL-8), the release of which recruits additional neutrophils to the site of inflammation or infection (43). After 60 min bacterial coincubation, we observed a clear reduction in IL-8 secretion by neutrophils exposed to WT GAS compared to the ΔSLO mutant (Figure 4B). Finally, the mean distance of neutrophil migration over 3 min in response to IL-8 as stimulus was significantly reduced in neutrophils exposed to WT GAS compared to those exposed to the ΔSLO mutant (Figure 4C). Thus, SLO production by GAS impairs neutrophil IL-8 release and their activation and migratory responses to the chemokine.

### SLO Inhibits Neutrophil Extracellular Trap Formation

**Figure 5:**
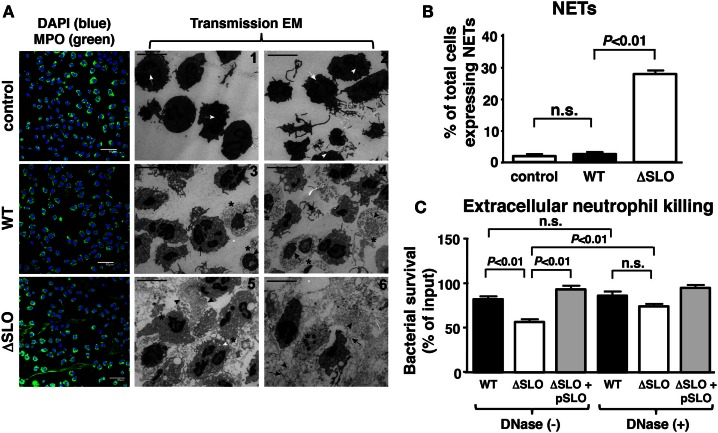
**Streptolysin O inhibits NET formation and extracellular killing**. **(A)** Representative images from NET induction with GAS are shown by immunofluorescence (IF) and transmission electron microscopy (TEM) for untreated control [white arrows = multi-lobulated nuclei; white arrowheads = granules; white arrows = multi-lobulated nuclei], GAS WT [asterisk = decondensed chromatin; arrowhead = disintegrated nuclear envelope; arrow = disrupted nuclear membrane; asterisk = decondensation of chromatin; arrowhead = disintegrated nuclear envelope; arrow = disrupted cell membrane] and ΔSLO mutant [arrow = dilated space between inner and outer membrane; arrowhead = cytoplasmic protrusions and vacuolization; white arrowhead = phagocytosed bacteria; asterisk = dissolved membrane and DNA; arrowhead = vesicles of globular proteins; arrow = fibrous DNA strands; asterisk = dissolved membrane], bar = 5 μm TEM. **(B)** SLO suppresses NET formation as quantified for GAS WT, ΔSLO mutant and media only control by IF. **(C)** Extracellular killing in the presence of cytochalasin D of GAS WT, ΔSLO and complemented strain shows that SLO to protect against killing in the presence/absence of DNase control to degrade NETs. Results are given in average ± SEM and analyzed by Student’s *t*-test (n.s., not significant).

Another critical innate immune function from neutrophils dependent on oxidative burst activity is NET formation, a specialized cell death process in which the nuclear DNA, embedded with antimicrobial histones and proteins, is released to ensnare and kill bacteria at tissue foci of infection (44, 45). Neutrophils were incubated with GAS WT and ΔSLO mutant at MOI of 1 bacteria/cell for 4 h then stained with antibodies against myeloperoxidase (MPO), the most abundant neutrophil granule protein that colocalizes within NETs (46). GAS SLO expression inhibited NETosis indicated by dissolved cell membranes and fibrous, extracellular DNA strands as determined by TEM and immunofluorescence (IF) microscopy (Figure 5A). The percentage of NET-releasing neutrophils was quantified by counting random IF microscopy fields (Figure 5B). M1T1 GAS strains also produce a nuclease (Sda1) that degrades the DNA backbone of NETs (9, 10). We confirmed that the GAS WT and ΔSLO mutant did not differ in their nuclease activity (Figure S3 in Supplementary Material). When neutrophils were treated with cytochalasin D to block phagocytic uptake of bacteria, survival of the GAS WT and complemented strain was greater than the survival of the ΔSLO mutant, differences that were abolished by DNase treatment (Figure 5C). This indicates that a DNA-dependent, extracellular killing process contributes to the neutrophil resistance phenotype provided by SLO expression.

### Inactivated SLO as a Protective Vaccine Antigen *In vivo*

**Figure 6:**
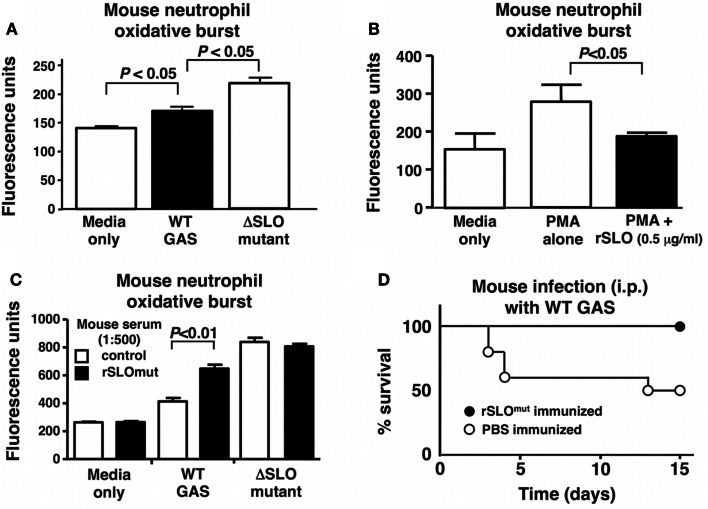
**Immunization with inactivated rSLOmut protects against GAS systemic infection**. SLO suppresses the oxidative burst in mouse neutrophils with **(A)** live GAS WT compared to ΔSLO mutant or **(B)** rSLO protein after PMA-stimulation including media alone as control. Serum from rSLOmut-immunized mice inhibits SLO suppression of neutrophil oxidative burst from live bacteria **(C)**. Immunization of mice with rSLOmut protein Vs mock prevents mortality of mice challenged with GAS WT in systemic infection model **(D)**. Results are shown as ±SEM from a minimum of three independent experiments. Data were analyzed by Student’s *t*-test.

Streptolysin O is a potent toxin that triggers cell apoptosis *in vivo* (47), and deletion of SLO from highly hemolytic M1 and M3 GAS strains (Figure 2A) has previously been associated with loss of virulence in murine models of systemic infection (19, 48). Potentially, due to GAS being a human-adapted pathogen, the oxidative burst response of human neutrophils was more robust than that of mouse neutrophils; nevertheless, we found a similar effect wherein murine neutrophil ROS production was significantly reduced upon exposure to WT GAS compared to the ΔSLO mutant (Figure 6A). Recombinant SLO also suppressed the oxidative burst response of PMA-stimulated murine neutrophils (Figure 6B). Native SLO may be a poor vaccine candidate because of its high cell toxicity, but strategies exist to mutate the cell-binding domain to create a toxoid SLO protein (rSLOmut) (32). Mice were immunized three times 2 weeks apart with rSLOmut, and a significant response in anti-SLO IgG and IgM antibodies was documented in serum by ELISA (Figure S4 in Supplementary Material). The post-immune antisera was found to reverse the inhibitory effect of SLO on the oxidative burst of mouse neutrophils (Figure 6C), demonstrating the capacity of the antibodies to block SLO neutrophil suppression. Mice immunized with rSLOmut were significantly protected against lethal systemic challenge with WT GAS M1 strain (Figure 6D), further supporting the inactivated SLO antigen as an attractive component for future multivalent GAS vaccines.

## Discussion

Group A *Streptococcus* remains one of the top 10 causes of infection-related morbidity and mortality worldwide (1). The aim of this study was to investigate the mechanisms GAS employs at early time points of infection (<30 min) to resist innate immune clearance by neutrophils. Generation of ROS upon bacterial encounter is an immediate neutrophil response to kill pathogens and to activate inflammatory signaling to recruit immune cells to the site of infection. As GAS is a non-pigmented and catalase-negative bacterium, it is dependent on alternative strategies to circumvent hostile ROS-rich host environments (11). Here, we identify SLO as the major bacterial factor responsible for suppressing the oxidative burst in neutrophils, facilitating GAS escape from innate immune killing. Previously, a study using the recombinant Mac-1/IdeS protease from GAS proposed that this virulence factor could be suppressing ROS production (49). However, physiological levels of Mac-1/IdeS from live bacteria had no effect on ROS response or virulence (50). To the best of our knowledge, SLO is the first GAS virulence factor shown to be necessary and sufficient for suppressing production of bactericidal ROS, thereby subverting neutrophil ROS-dependent killing. Our study shows that SLO produced by live bacteria as well as recombinant SLO protein is necessary and sufficient to modulate innate immune cell functions at early time points of infection. A specific suppression of oxidative burst from neutrophils by SLO was further demonstrated by using specific inhibitor of SLO activity, cholesterol, or anti-SLO blocking antibodies.

Streptolysin O, PLY, LLO, and PFO, all belong to the same family of CDCs (20). Interestingly, neither the pore-forming staphylococcal α-hemolysin nor its production from live *S. aureus* had a similar effect on oxidative burst in neutrophils, suggesting a specific unique role of SLO and related toxins that is not a general consequence of membrane perturbation and disruption. Recently, LLO and PFO have been shown to suppress oxidative burst in murine macrophages by preventing the localization of NADPH oxidase with the phagosome (51).

To extend the analysis of SLO in suppressing neutrophil functions, we investigated its role in additional ROS-dependent functions critical in clearance of bacterial pathogens. The production of ROS is linked to degranulation and the elaboration of extracellular traps, which is a critical function for entrapment and extracellular killing of bacteria (44). Our findings show that SLO prevents the release of IL-8 and elastase from neutrophils and blocks the formation of NETs. In our study, we confirmed that SLO contributes to resistance to an extracellular killing mechanism that is DNA-dependent. The hyperinvasive M1T1 GAS clone expresses a potent phage encoded nuclease (Sda1) that, like SLO, is upregulated on *cov*R/S mutations arising *in vivo* under innate immune selection (15, 16). Thus, GAS has evolved a dual-pronged approach to both reduce the production of NETs, and to further dissolve NETs that are established, effectively neutralizing this arm of innate immunity.

In summary, we described a number of incapacitating effects of SLO on neutrophil function that act rapidly to suppress neutrophil function in advance of the toxin’s ultimate and previously established role in triggering cell death pathways in the phagocyte. The importance of these virulence phenotypes is underscored by the protective effect of active immunization with an rSLOmut toxoid vaccine against invasive GAS infection. As SLO is present in all GAS serotypes and strains, our findings support proposals (3, 32) to include inactivated SLO toxoid as a component of a multicomponent vaccine against a leading human pathogen, GAS.

## Conflict of Interest Statement

The authors declare that the research was conducted in the absence of any commercial or financial relationships that could be construed as a potential conflict of interest.
