# Supplementary material for: Streptolysin O Rapidly Impairs Neutrophil Oxidative Burst and Antibacterial Responses to Group A Streptococcus
Source: Front Immunol. 2015 Nov 16;6:581. doi: 10.3389/fimmu.2015.00581 (PMC4644796; doi:10.3389/fimmu.2015.00581)
Supplement: Supplementary file 1 [file Data_Sheet_1.PDF]

Supplemental Materials

Streptolysin O Rapidly Impairs Neutrophil Oxidative Burst and Antibacterial Responses to Group A *Streptococcus*

Satoshi Uchiyama, Simon Döhrmann, Anjuli M. Timmer, Neha Dixit,
Mariam Ghochani, Tamara Bhandari, John C. Timmer, Kimberly Sprague,
Juliane Bubeck-Wardenburg, Scott I. Simon, Victor Nizet

Contents:

Supplemental Methods and References

Supplemental Figures S1-S4 with legends

SUPPLEMENTARY METHODS

Bacterial Strains

GAS Wild-type (WT) strain M1T1 5448 was originally isolated from a patient with necrotizing fasciitis and toxic shock syndrome (Chatellier et al., 2000). GAS WT, ΔSLO mutant and complemented (ΔSLO+pSLO) strain were created previously (Timmer et al., 2009). Methicillin-resistant *S. aureus* (MRSA) USA 300 TCH1516 WT, Δhla mutant and complemented (Δhla+phla) strain were used. Bacteria were cultivated in Todd-Hewitt broth (THB) at 37°C.

Cell viability

Neutrophil viability is either determined by Live-Dead staining or LDH release upon infection at MOI of 1, 10 and 50 after 30 min infection with live GAS. For LDH release the cell viability was measured according to manufacture's instructions with CytoTox 96 non-radioactive cytotoxicity assay (Promega). Briefly, 2 x 10^5 neutrophils were infected with GAS M1 WT, ΔSLO, ΔSLO+pSLO strain at an MOI of 1, 10 and 50. For maximal release of LDH, neutrophils were lysed with 1% Triton X-100 (positive control) or incubated in HBSS with Ca^{2+} and Mg^{2+} (negative control). The absorbance was determined after 30 min incubation at RT on SpectraMax M3 plate reader at 490 nm for LDH release using SoftMax

Pro software. In addition, we analyzed cell viability by staining with a Live-Dead staining (Invitrogen). Neutrophils were infected with live GAS WT and ΔSLO at an MOI of 1 for 30 min and stained for 20 min with Live-Dead staining, analyzed by immunofluorescence microscopy and quantified with ImageJ software. Representative images are shown.

Oxidative burst assay

Oxidative burst assays were performed as previously described (Chow et al., 2009). Briefly, 2 x 10^6/mL neutrophils were resuspended in HBSS without Ca^{2+} and Mg^{2+} and incubated rotating with 20 µM 2,7 DCFH-DA at 37°C for 20 min. Neutrophils were resuspended to 2 x 10^6 cells/mL. 2 x 10^5 cells/well were infected at an MOI of 1 and 10 with Methicillin-resistant *S. aureus* TCH1516 WT, Δhla and complemented strain (Δhla+phla) for 20 min. Assays were performed with 25 nM PMA as positive control, active and inactivated recombinant SLO protein and α-hemolysin from *S. aureus* (Sigma) as stated. The fluorescence intensity was quantified on a SpectraMax M3 plate reader at 485nm ex/520nm em using SoftMax Pro software.

DNA degradation assay

To test the nuclease activity from live GAS bacteria, 2 x 10^5 CFU were incubated with 1 µg/well lambda-DNA provided from Quant-iT PicoGreen (Invitrogen) for 4 h. 10 min prior

quantification 500 mU/mL DNase 1 (Sigma) was added to samples as a positive control. Media alone acts as a negative control. The supernatant containing DNA was incubated with Quant-iT PicoGreen according to manufactures instructions. The fluorescence intensity correlates with amount of dsDNA and was quantified on a SpectraMax M3 plate reader at 480 nm ex/520 nm em using SoftMax Pro software.

Anti-SLO IgG and IgM antibody in mouse serum by ELISA

Mice were immunized with rSLOmut or mock for three times two–weeks apart. Immulon^{TM} 4HBX 96-well plate (Thermo Electron) were coated O/N at 4°C with 500 ng/mL purified SLO protein in carbonate buffer (pH 9.4). Wells were washed with TBST (50mM Tris-Hcl, 150mM NaCl, 0.1% Tween-20), blocked for 1 h at RT with TBST and incubated with mice serum (1:100 diluted) for 3 h at RT. For detection an AP-conjugated goat-anti mouse IgG antibody (Promega) was used and incubated with p-nitrophenyl phosphate for 1 h. Anti-SLO antibody titer was determined via absorbance at 405 nm with SpectraMax M3 plate reader using SoftMax Pro software.

Statistical analysis

All data were collected from at least three independent experiments in triplicate. Experiments using neutrophils were performed with a minimum of three different healthy volunteers. The

data were combined and expressed as mean ± SEM except stated differently. All data were analyzed by unpaired Student's t-test using GraphPad Prism version 5.0 (GraphPad Software Inc.). P values < 0.05 were considered statistically significant.

REFERENCES

Chatellier, S., Ihendyane, N., Kansal, R.G., Khambaty, F., Basma, H., Norrby-Teglund, A., Low, D.E., Mcgeer, A., and Kotb, M. (2000). Genetic relatedness and superantigen expression in group A streptococcus serotype M1 isolates from patients with severe and nonsevere invasive diseases. Infect Immun 68, 3523-3534.

Chow, O.A., Von Köckritz-Blickwede, M., Bright, A.T., Hensler, M.E., Zinkernagel, A.S., Cogen, A.L., Gallo, R.L., Monestier, M., Wang, Y., Glass, C.K., and Nizet, V. (2009). Statins enhance formation of phagocyte extracellular traps. Cell Host Microbe 8, 445-454. doi: 10.1016/j.chom.2010.10.005.

Timmer, A.M., Timmer, J.C., Pence, M.A., Hsu, L.C., Ghochani, M., Frey, T.G., Karin, M., Salvesen, G.S., and Nizet, V. (2009). Streptolysin O promotes group A *Streptococcus* immune evasion by accelerated macrophage apoptosis. J Biol Chem 284, 862-871. doi: 10.1074/jbc.M804632200.

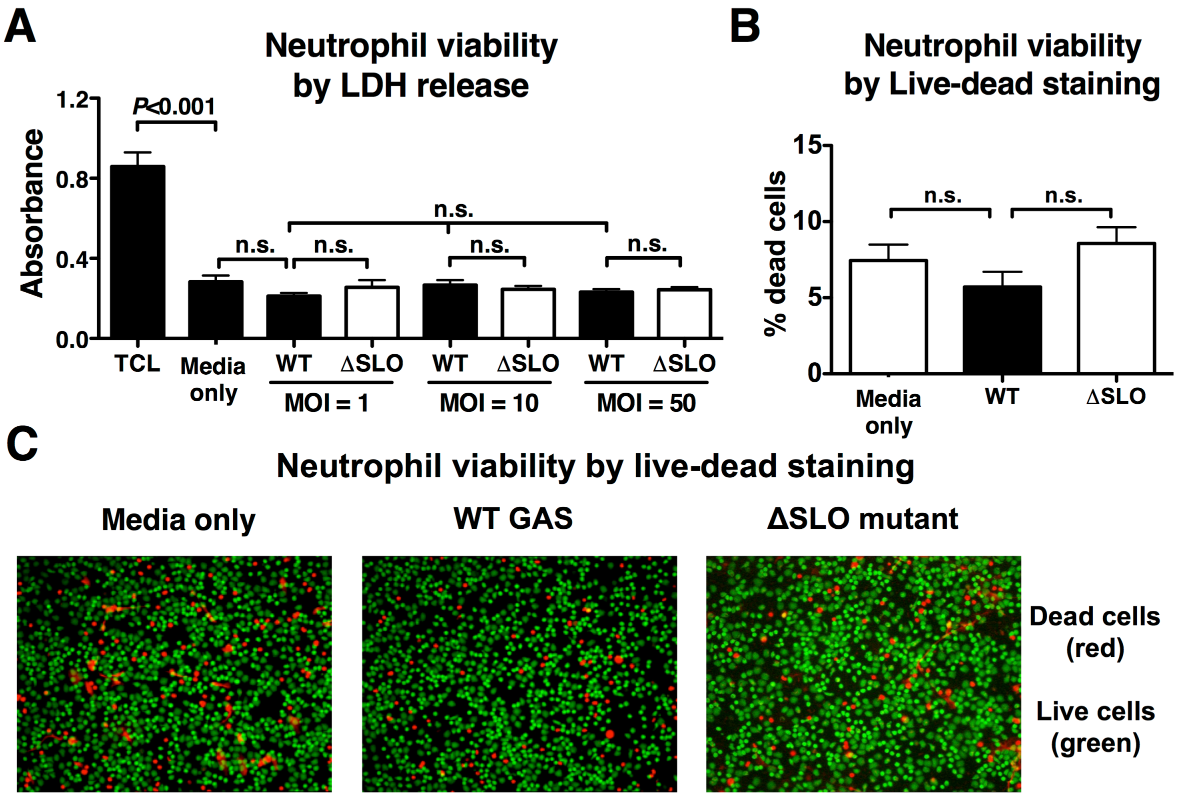

Supplemental Figure S1. **Lack of SLO cytotoxicity at early time points under study.** SLO expressed in WT GAS bacteria does not induce accelerated cytotoxicity in neutrophils at early time points as determined by (A) LDH release and (B) Live-dead staining. (**C**) Representative images from Live-Dead staining were shown. Results are given in average ± SEM and analyzed by Student's t-test (n.s.,= not significant). Control denotes no treatment.

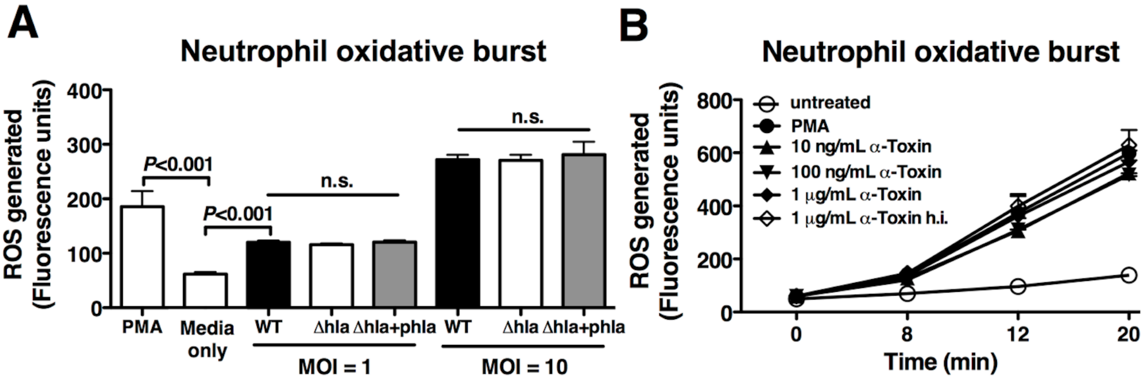

Supplemental Figure S2. ***Staphylococus aureus*** **alpha-toxin does not block neutrophil oxidative burst.** Neutrophil oxidative burst was not affected by (**A**) live S. aureus WT and ΔHla mutant at MOI of 1 and 10 and (**B**) recombinant protein at 10-1000 ng/mL. Results are given in average ± SEM and analyzed by Student's t-test (n.s., not significant, P<0.01 and P<0.001).. Control denotes no treatment.

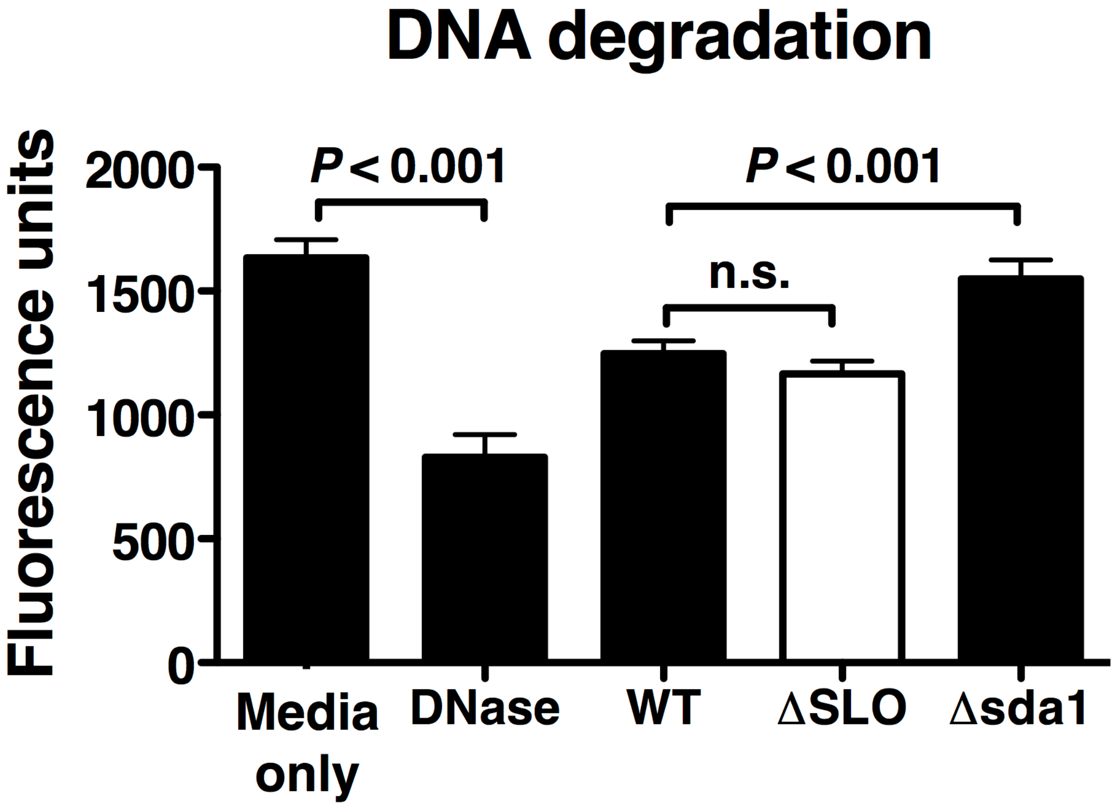

Supplemental Figure S3. **Nuclease activity is not altered in the GAS ΔSLO mutant.** (**A**) Nuclease activity was not changed in GAS ΔSLO mutant compared to WT as compared to nuclease-deficient Δsda1 mutant, DNase 1 and media over 4 h as quantified by Quant-iT Picogreen kit. Results are given in average ± SEM and analyzed by Student's t-test (n.s., not significant and P<0.001). Control denotes no treatment.

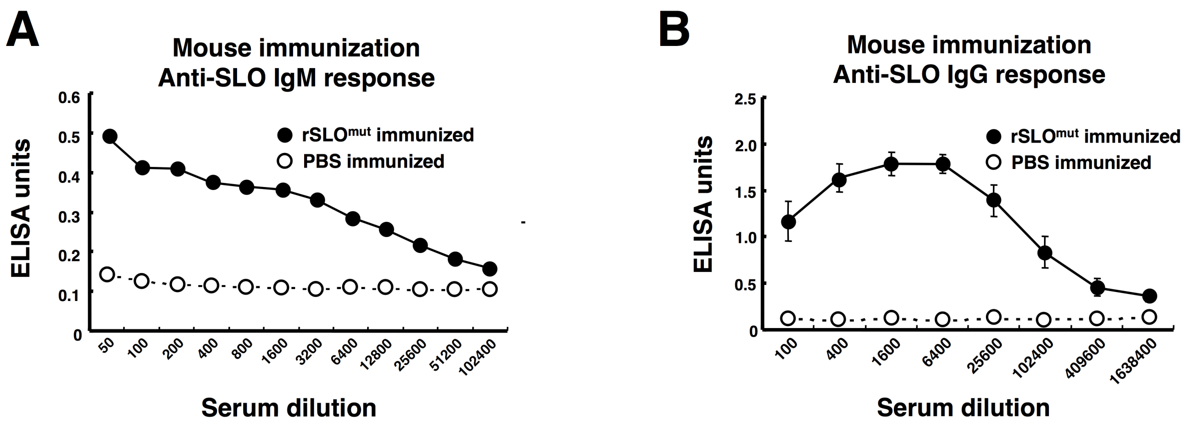

Supplemental Figure S4. Anti-SLO^{mut} antibody titers from mice following active immunization with a recombinant SLO^{mut} protein. Anti-SLO titers were determined after three immunizations with recombinant SLO^{mut} for (**A**) IgM and (**B**) IgG response by ELISA.
